# Employment of Chloride of Zinc in Toothache

**Published:** 1844-06

**Authors:** 


*Employment of Chloride of Zinc in Toothache.* By Dr. Stanelli.—According to Dr. Stanelli, the chloride of zinc, liquified by exposure to the air, possesses the property of calming dental pains.

His mode of application is most simple. By means of a small hair pencil, a small quantity of it is applied to the cavity of the painful tooth, and in the space of a few minutes it appeases the most acute sufferings, without causing any irritation.

Before proceeding to the application, it is indispensable carefully to surround with cotton wadding, and, when the chloride has been applied, to well fill the cavity with this same cotton. The mouth is finally washed with a little warm water.

The author affirms that he has obtained uniform success from this means in more than fifty cases, and that he has never observed the progress of the caries rendered more active by it.—*Annali Universali de Medicina; and Chemist.*

